# Supplementary material for: The genome sequence of the Pearly Heath, Coenonympha arcania Linnaeus, 1761 (Lepidoptera: Nymphalidae)
Source: Wellcome Open Res. 2025 Jul 25;10:364. [Version 1] doi: 10.12688/wellcomeopenres.24624.1 (PMC12461188; doi:10.12688/wellcomeopenres.24624.1)
Supplement: Peer review history

# Reviewer report: Reviewer response for version 1

**DOI:** 10.21956/wellcomeopenres.27135.r132655

**Published:** 2025-09-25

**Authors:** Quentin Rougemont

**Affiliations:** 1 Université Paris Saclay, Paris, France

**Review of** **'The genome sequence of the** **Pearly Heath** **,** ***Coenonympha arcania*** **Linnaeus, 1761** **(Lepidoptera: Nymphalidae)**

 This is a high-quality genome assembly for the Pearly Heath, providing providing valuable ressources for future studies, especially given the existence of other genome in this species complex.

 The genome assemblies was easily accessible online, which is great.

 The whole pipeline follows standard practice and the report of the results meet the quality requirement. The pipeline is also easilly findable online following the provided links.

 The merian plot was really nice.

 I am a bit surprised by the very low k-mer completeness of each haplotype assembly separately. Is this due to a high number of structural variation between each haplotype ?

 Minor comments:

 It would be nice to specify whether only default parameters were used for each tools or if some parameters had to be set to some specific values.

 Why not showing busco scores from the ‘lepidoptera_odb12’, moreover using the BUSCO6.0.0 seems to provide more accurate identification of busco genes, compared with the BUSCO5.5.0 used here.

 Was there any evidence of contamination from the ASCC pipleine?

Are sufficient details of methods and materials provided to allow replication by others?

Yes

Is the rationale for creating the dataset(s) clearly described?

Yes

Are the datasets clearly presented in a useable and accessible format?

Yes

Are the protocols appropriate and is the work technically sound?

Yes

Reviewer Expertise:

population genomics

I confirm that I have read this submission and believe that I have an appropriate level of expertise to confirm that it is of an acceptable scientific standard.

# Reviewer report: Reviewer response for version 1

**DOI:** 10.21956/wellcomeopenres.27135.r132654

**Published:** 2025-09-24

**Authors:** Jasmine D. Alqassar

**Affiliations:** 1 The George Washington University, Washington, District of Columbia, USA

In this article Vila et al. present a high-quality chromosome-level reference genome for *Coenonympha arcania*, the pearly heath butterfly. BUSCO analysis demonstrates the genome assembly is highly contiguous and complete (BUSCO score: C: 98.2%[S:97.7, D:0.5%], F: 0.34%, M:1.76% n=5286). This genome resource will serve as a valuable resource for the lepidopteran genomics community.

 Although it is possible that this species does not have a W chromosome and instead uses the ancestral ZZ/ZO sex determination system, a lack of a W chromosome in a genome assembly is only partial evidence of this possibility because of the known difficulties associated with sequencing lepidopteran W chromosomes. Further cytological analysis would be needed to confirm the true absence of a W chromosome. Therefore, I ask that the authors amend the latter half of the following statement in the “Assembly statistics” section “There was no evidence of a W chromosome in this assembly, therefore it appears that this species exhibits the ZO karyotype.” to instead state that “this suggests that this species may exhibit the ZO karyotype; however this possibility must be confirmed by further cytological analysis”.

Are sufficient details of methods and materials provided to allow replication by others?

Yes

Is the rationale for creating the dataset(s) clearly described?

Yes

Are the datasets clearly presented in a useable and accessible format?

Yes

Are the protocols appropriate and is the work technically sound?

Yes

Reviewer Expertise:

Genomics and evolutionary developmental biology

I confirm that I have read this submission and believe that I have an appropriate level of expertise to confirm that it is of an acceptable scientific standard.

# Reviewer report: Reviewer response for version 1

**DOI:** 10.21956/wellcomeopenres.27135.r130506

**Published:** 2025-09-09

**Authors:** Fedor Čiampor

**Affiliations:** 1 Slovak Academy of Sciences, Bratislava, Slovakia

This report focuses on generating the genome of Pearly Heath *Coenonympha arcania* Linnaeus, 1761, a small butterfly from the Nymphalidae family. The reason for sequencing the genome of this species is to study its position in the species complex and also the role of hybridization and gene flow in the diversification and adaptation of the clade.

 Although I am not very proficient in WGS, in my opinion, the methods and outputs were described very thoroughly and accurately.

 The only comment I have on the report is why did the authors use a female? In insect taxonomy, it is very common that females are very difficult to determine, and species are very often clearly delimited based only on male genitalia. Furthermore, if the sequenced species belongs to a complex in which species hybridize with each other, how can the authors be 100% sure that the species they sequenced is *Coenonympha arcania*? Perhaps in this case there is evidence that the female used does indeed belong to the species mentioned, but it would be good to mention it.

 And, please check the sentence "Normalised libraries were quantified again and equimolar and/or weighted 2.8 nM pools.". It doesn't sound quite right to me.

Are sufficient details of methods and materials provided to allow replication by others?

Yes

Is the rationale for creating the dataset(s) clearly described?

Yes

Are the datasets clearly presented in a useable and accessible format?

Yes

Are the protocols appropriate and is the work technically sound?

Yes

Reviewer Expertise:

DNA (meta)barcoding, biodiversity, taxonomy, phylogeny insects, mostly freshwater beetles

I confirm that I have read this submission and believe that I have an appropriate level of expertise to confirm that it is of an acceptable scientific standard.
